# Effect of Starch Variety and Environmental Conditions on the Aerobic Biodegradation of Citric Acid-Compatibilized Thermoplastic Starch/Polylactic Acid Blends

**DOI:** 10.3390/polym17101295

**Published:** 2025-05-08

**Authors:** Elizabeth Moreno-Bohorquez, Mary Judith Arias-Tapia, Keydis Martínez-Villadiego, Jesús D. Rhenals-Julio, Andrés F. Jaramillo

**Affiliations:** 1 Chemical Engineering Program, School of Engineering, Universidad Tecnológica de Bolívar, Parque Industrial y Tecnológico Carlos Vélez Pombo km 1 Vía Turbaco, Cartagena 130010, Colombia; morenoe@utb.edu.co (E.M.-B.); kmartinez@utb.edu.co (K.M.-V.); 2 Departamento de Ingeniería Mecánica, Universidad de Córdoba, Cr 6 #76-103, Montería 230002, Colombia; jesusrhenalsj@correo.unicordoba.edu.co; 3 Department of Mechanical Engineering, Universidad de La Frontera, 01145 Francisco Salazar, Temuco 4780000, Chile

**Keywords:** biodegradability, PLA, TPS, compost, seawater, *Dioscorea rotundata*, *Ipomoea batatas*

## Abstract

In this study, the aerobic degradation of sweet potato (*Ipomoea batatas*; *SP*) and diamond yam (*Dioscorea rotundata*; *DY*) thermoplastic starch (TPS) blends, combined with polylactic acid (PLA) and varying ratios of citric acid (CA) as a crosslinker, was investigated in compost and seawater environments. After 50 d of composting, weight losses in the SP-TPS/CA/PLA blends were 56.9%, 52.3%, and 77.5%, while those of DY-TPS/CA/PLA were 55.8%, 52.2%, and 62.2% for 0%, 1%, and 5% CA, respectively. In seawater, the SP-TPS/CA/PLA blends showed weight losses of 52.9%, 46.8%, and 61.5%, and the DY-TPS/CA/PLA blends lost 35.2%, 32.1%, and 43.9% for the same CA ratios, respectively. In both media, SEM revealed structural damage, holes, cracks, and changes in coloration, reflecting microbial activity. Additionally, in compost and seawater, TGA results showed that PLA remained the predominant component after 50 d, as most of the degradation occurred on TPS due to its amorphous structure and higher hydrophilicity. In both media, the SP-TPS/CA5/PLA and DY-TPS/CA5/PLA blends exhibited faster degradation, whereas SP-TPS/CA1/PLA and DY-TPS/CA1/PLA displayed higher stability and lower disintegration. Additionally, all blends required over 50 d to degrade completely, as evidenced by the absence of a plateau phase in the biodegradability curves. Statistical analysis showed that, in seawater, the degradation behavior of the blends was similar to cellulose. However, the CA ratio had a greater impact on the compost degradation of the blends with SP-TPS than on DY-TPS. Therefore, the critical factors influencing the degradation of these blends are the starch source and the CA ratio.

## 1. Introduction

Owing to current societal demands and their diverse applications in many fields, such as technology, medicine, and food, plastics are one of the largest sources of industrial production [1]. The potential of these materials is mainly attributed to their versatility, low production costs, and excellent mechanical and thermal properties owing to their structural distribution [2]. However, the high demand for these products has generated a significant amount of waste because they are not adequately managed at the end of their useful lives [3]. Their disposal poses a major environmental problem because they take several decades to fully degrade, thereby generating pollution. According to Rhodes [4], only 9% of the global plastic production has been recycled. Moreover, the percentage of incineration of these plastics did not exceed 12%, which is not even a quarter of the more than 8 billion tons of plastic generated between 1950 and 2015. The incineration of these products results in substantial amounts of CO_2_ being released into the atmosphere, which does not help reduce greenhouse gas emissions [3].

Plastic pollution harms all marine species in various ways, including plastic ingestion, entanglement in debris, and exposure to toxins released during degradation [5]. Every day, tons of plastic are discarded worldwide in all types of ecosystems, to the extent that the amount of waste present in the marine environment is unknown [6]. The main problem with plastic waste lies in its high toxicity during degradation, releasing toxins mainly from the additives used to improve its properties at the time of manufacture (such as phthalates, styrene oligomers, perfluorinated compounds, etc.), which are subsequently absorbed by marine fauna [5].

Therefore, alternative polymeric sources that are more sustainable and exhibit shorter degradation times than conventional plastics are crucial [7,8,9]. For example, Singh et al. [10] reported that only 1% of the entire plastic production is composed of bioplastics, wherein biodegradable materials constitute 54% of this small percentage, with PLA making up approximately 20% of the main component in the bioplastics list.

PLA is a polymer obtained through the polymerization of lactic acid, which is naturally derived from saccharides such as maltose, glucose, sucrose, and even natural polymers such as starch [11]. This material has gained importance over the last few decades due to its mechanical, thermal, and barrier properties [12]. These characteristics facilitate the manufacture of products that are normally produced from fossil-based plastics [13], thus making them a viable alternative to address the issue of white pollution. However, their production costs are high [14], which is a disadvantage compared to conventional plastic manufacturing. Furthermore, although PLA degrades faster, the process can still take around six months to two years and potentially take longer in cases where this material has high crystallinity and is exposed to low environmental temperatures [11]. Hence, this material can be blended with other low-cost materials that degrade it more easily and reduce overall production costs, without significantly compromising the typical properties of petroleum plastics [7,8].

Starch is a polysaccharide that is abundant in tubers and can also be found in many other plant sources [14]. Its production is cheap, and it has a high level of biodegradability due to its organic origin. However, this component is highly hydrophilic and exhibits uncompetitive properties compared to fossil-based plastics (polyethylene terephthalate, polypropylene, etc.) [15]. Previous studies have demonstrated that processing starch with plasticizing agents, such as glycerol, water, or sorbitol [16,17], can reduce its intermolecular forces, enabling a transition from a crystalline to an amorphous structure that enhances its thermoplastic behavior [14,18]. Although TPS offers better mechanical properties compared to native starch, the presence of OH- groups makes it hydrophilic [18], which can be considered a disadvantage. Additionally, the mechanical properties of this material (such as tensile stress, elongation at break, and water resistance) are limited and poor in comparison to those of conventional plastics [14,15], which makes its development as a sustainable alternative to address the abovementioned problems unlikely.

Therefore, the integration of this biodegradable component into commercial synthetic polymers, which would lower degradation times and reduce environmental pollution, has been previously studied [14,18]. However, due to the chemical differences between the components, the resulting blends are incompatible. To address this, an additional substance that allows the interfacial interaction of all the components is required. Carboxylic acids are effective compatibilizing agents for plastic blends with TPS. In addition to being derived from renewable sources [18,19,20], these acids are organic compounds with polar functional groups that interact well with the TPS group, thereby enhancing interfacial adhesion via chemical crosslinking and promoting biodegradability. This interaction can provide the blend with enhanced properties for processing as commercial plastic (melting point, degradation temperature, etc.) [18]. Previous studies have evaluated the effects of CA on PLA and TPS/PLA blends, achieving improvement by increasing the degree of interaction, inhibiting retrogradation, and enhancing their thermal properties [21,22,23].

A study conducted by Muniyasamy et al. [24] evaluated the aerobic degradation of poly (3-hydroxybutyrate-co-3-hydroxyvalerate) (PHBV), PLA, and PHBV/PLA blends in respirometric flasks, monitoring the evolution of CO_2_ released during decomposition. After 200 d, pure PLA blend exhibited a biodegradability of 10% in a composting environment. These results proved that although PLA is considered a biodegradable material, it requires more than six months to achieve significant degradation [11]. Ibrahim et al. [22] studied the degradation of PLA/TPS blends with 0–6% CA compositions over a two-month burial period, obtaining greater degradation in the blends with higher CA content. Notably, this blend could degrade over 60 d, which was more than twice than pure PLA at 200 d, as reported by Muniyasamy et al. [24].

Sweet Potato (SP) and Diamond Yam (DY) are highly available local tubers in the Montes de Maria region (Colombia), where agricultural overproduction has led to surplus and crop losses [25]. These crops offer alternative uses such as starch feedstocks for bioplastics [26], and limited research has been conducted on the use of blends incorporating these starches. Therefore, the aim of this study is to evaluate the effect of CA on the degradation of TPS/PLA blends containing SP and DY starches in compost and seawater, through degradative, thermal, and morphological characterization.

## 2. Materials and Methods

### 2.1. Raw Materials

The TPS/PLA blends were compatibilized using CA at ratios of 0%, 1%, and 5% and were processed according to the methodology and conditions employed by Martínez Villadiego [27]. Ba(OH)_2_, phenolphthalein, HCl, commercialized by Merck Millipore (Darmstadt, Germany) and HANNA Instruments^®^ (Woonsocket, RI, USA) seawater analysis tests were used in this study. The sandy sediment and seawater were taken from Bocagrande Beach (10°24′08″ N–75°33′31″ W, Cartagena, Colombia). ERGO^®^ perlite substrate (Düsseldorf, Germany) and FORZA^®^ compost (Yumbo, Colombia) were used. Finally, a PLA compostable bag MAHIZ^®^ (Sabaneta, Colombia) was used as a control, and cellulose filter paper Whatman^®^ (Buckinghamshire, UK) and polyethylene (PE) bags (Esenttia, Cartagena, Colombia) were used as positive and negative references, respectively.

### 2.2. Preparation and Conditioning of TPS/CA/PLA Blends

The TPS/CA/PLA blends were obtained from a previous study [27], and their compositions are listed in Table 1. Compatibilizer concentrations were selected based on the literature indicating that levels below <5% enhance film strength through crosslinking, while higher concentrations act as plasticizers and reduce mechanical performance [28]. The TPS/CA/PLA blends were laminated by heating the blends with a thermal blower (SKIL) at approximately 180 °C until they were deformed. Subsequently, they were placed into a 4 cm^2^ plasterboard mold and pressed with a 1 kg body until they reached 25 °C. Once cooled, the samples were removed from the mold and hermetically stored in polypropylene bags. Prior to the testing, the blends were treated according to the ASTM D618 standard (27.6 ± 2 °C, relative humidity of 63 ± 10%) for 48 h. The experimental conditions were conducted to rule out possible abnormal conditions that might affect the outcome, as required by the standard. Additionally, a sample of each of the blends was extracted to determine the volatile solid content by drying in an oven (HUMBOLTD, IL, USA) at 105 °C and subsequent heating in a muffle Paragon (Wensterville, OH, USA) at 550 °C. The total organic carbon (TOC) percentages of the blends were obtained using Equation (1), proposed by Junior et al. [29].

(1) $$TOC=0.425\left[\mathrm{Volatile}\;\mathrm{Solids}\;\mathrm{Content}\right]-2.064$$

### 2.3. Degradation of TPS/CA/PLA Blends in Compost

Compost was initially sieved through a 3-mesh sieve, according to the methodology proposed by Nomadolo et al. [30], and part of it was heated in a convection oven (HUMBOLDT) at 105 °C and 101,325 Pa. The percentage of dry solids was determined as the ratio of the compost remaining in the container after drying to its initial mass. The remaining fresh compost was incinerated in a muffle (Paragon, Wensaterville, USA) at 550 °C and 1 atm for 6 h. After calcination, the volatile solids content was calculated as the proportion of mass lost during incineration relative to the initial mass subjected to combustion.

**Figure 1:**
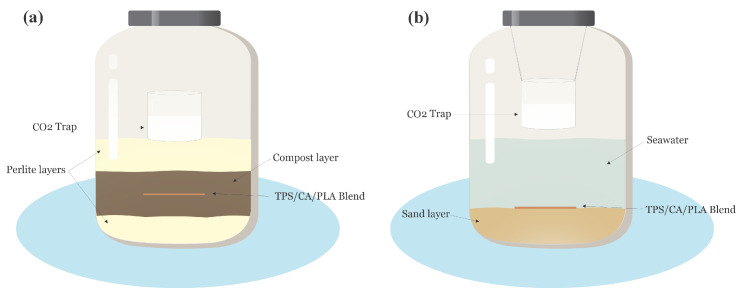
(**a**) Compost degradation setup with layered perlite, compost, and CO_2_ trapping. (**b**) Seawater–sand interface assembly for aerobic degradation monitoring.

The experimental setup was carried out according to ASTM D6400 and the methodology proposed by Nomadolo et al. [30]. Glass jars with lids were used to deposit 15 g of the perlite substrate, which was previously moistened with 15 mL of distilled water, in a uniform layer, as shown in Figure 1a [31]. The TPS/CA/PLA blends were introduced in a 6:1 compost-to-blend ratio. An additional 15 g moisturized perlite layer was placed on top of the compost, and a 0.025 N Ba(OH)_2_ (ac) container was used to measure the released CO_2_ during degradation.

The blends were incubated for 50 d, with periodic monitoring every 3 or 4 d during the first four weeks, to prevent exceeding the CO_2_ absorption capacity of the Ba(OH)_2_ container. Subsequently, monitoring was performed weekly until the end of the incubation period. Each monitoring cycle involved extracting the blends, removing moisture with heat, weighing them, reassembling the experimental setup after remoisturizing the perlite, and placing fresh Ba(OH)_2_ for CO_2_ adsorption (the previous one was used for acid–base titration to quantify the CO_2_ absorbed).

### 2.4. Degradation of TPS/CA/PLA Blends in Seawater Environment

Seawater and sandy sediment were collected from the infralittoral zone of Bocagrande Beach, Cartagena, Colombia (10°24′08″ N–75°33′31″ W). Seawater density was measured in triplicate using a 25 mL glass pycnometer (Boeco, Germany). Salinity was determined using a hydrometer CORALIFE, Deep Six (Franklin, USA) and the mercuric nitrate titration method described in the HI 3835 procedure. TOC was measured using the purge method (Hach 10129) for a 1 L seawater sample. Alkalinity was estimated according to the total alkalinity method outlined in the HI 3811 method (ASTM D1067-16).

Dissolved carbon dioxide was quantified by converting the available carbonic acid in the sample to sodium bicarbonate using a dilute sodium hydroxide solution, as described in the HI 3818 method (ASTM D513-16). Nitrite, nitrate, and phosphate concentrations were measured using colorimetric methods, as proposed in the HI 3873, HI 3874 (ASTM D3867-16), and HI 3833 procedures, respectively. Dissolved oxygen was determined using a modified Winkler method following the HI 3810 method (ASTM D888-18). pH was measured in triplicate using a pH meter (HI 98127) to ensure accuracy (ASTM D1293-18). Conductivity was measured following the S.M. 2510-B method proposed by the Institute of Hydrology, Meteorology, and Environmental Studies (*IDEAM, by its acronym in Spanish*) for a 1 L water sample.

Glass flasks were used according to the ISO 19679 standard, wherein 70 mL of seawater and sandy sediment were added at a 5:1 ratio. The TPS/CA/PLA blends were placed at the water–sediment interface. Subsequently, a 0.025 N Ba(OH)_2_ container was placed, as shown in Figure 1b. Cellulose and PE were used as positive and negative references, respectively. PLA was used as a blank, and a flask without blends was used as control. The experimental setups were checked using the procedure described in Section 2.5 [32].

### 2.5. Biodegradability Assessment

Biodegradability was determined in terms of the amount of released CO_2_, following the ASTM D5338 and ISO 19679 standards [32,33]. The Ba(OH)_2_ contained in the vial was titrated with HCl in the presence of phenolphthalein until neutralization. The CO_2_ produced during degradation reacted with Ba(OH)_2_, as represented in Equation (2) [29,30]. This equation relates the number of Ba(OH)_2_ moles reacted to the mass of CO_2_ released by the sample using stoichiometry. The theoretical CO_2_ production was estimated using the TOC, as shown in Equation (3), where *m* is the mass of the blend, and *TOC* represents the total organic carbon content. Finally, biodegradability was determined using the ratio of CO_2_ to theoretical CO_2_. However, since the degradation media contains microorganisms that also produce CO_2_ endogenously, a correction was applied using the CO_2_ measured in the control flask, as shown in Equation (4) [29,30].

(2) $$Ba{\left(OH\right)}_{2}+CO_{2}\to BaCO_{3(s)}+H_{2}O$$

(3) $${CO}_{2}T=\left[m\right]\left[TOC\right]\left[44/12\right]$$

(4) $$Biodegradability\left(\%\right)=\left[\frac{CO_{2(test)}-CO_{2(control)}}{CO_{2}T}\right]$$

### 2.6. Morphological Characterization

TPS/CA/PLA blends were coated with a gold layer to produce conductive samples and subsequently analyzed in a JEOL JSM 6060 SEM scanning electron microscope to analyze their morphology. Micrographs were taken employing an accelerating voltage of 15 kV for 50 mg blends, before and after the degradation process [26].

### 2.7. Thermal Characterization

A TGA 5500 thermobalance (TA Instruments, New Castle, DE, USA) was used, and the weight of each blend was approximately 60 mg. Measurements were performed at a heating rate of 10 °C/min, from room temperature up to 600 °C. Each blend was kept in an inert atmosphere under a continuous flow of nitrogen at 100 mL/min to avoid thermo-oxidative reactions [26].

### 2.8. Preparation of Blends and Environment Characterization

DY (DY-TPS/CA/PLA) and SP (SP-TPS/CA/PLA) blends with varying CA ratios were developed based on our previous research using a torque rheometer. These blends were used to evaluate their biodegradation in two different media.

All blends were incubated in the compost and sand–seawater interface (see Figure 1) for 50 d. Blends were initially removed from the experimental setup weekly until the incubation period ended, then carefully dried, weighted, and taken back to the respirometry flasks. The physicochemical properties of compost and seawater are presented in Table 2 and Table 3, respectively.

### 2.9. Data Analysis and Processing

#### 2.9.1. Design of Experiments

Six categorical experimental treatments were designed using different combinations of starch types, polylactic acid (PLA), and citric acid (CA) as a compatibilizer. Biodegradability and mass loss percentages were evaluated at 5-to-9-day intervals over the degradation period. The experiments were conducted in two degradation media: compost and seawater. Each treatment was replicated three times to ensure reproducibility and to allow assessment of experimental variability. Table 4 presents the factors included in the experimental design, along with their respective levels and coded variables.

#### 2.9.2. Analysis of Variance

Statistical analyses were performed using a significance level of 5% (*p* < 0.05). An analysis of variance (ANOVA) was conducted to evaluate the individual and interactive effects of CA concentration, starch type, and degradation medium on the biodegradation performance of the materials. To examine the temporal evolution of degradation, two sets of ANOVAs were performed for each response variable: one at 21 days and another at 50 days. In each case, *p*-values were calculated to determine the statistical significance of the main effects and interactions. Where appropriate, standard deviations were computed and represented as error bars in the figures to reflect data dispersion and support interpretation of statistical significance.

#### 2.9.3. Tukey’s Multiple Comparisons Test

To identify statistically significant differences among the six formulated blends and the three control materials (PLA, PE, and cellulose), Tukey’s Honestly Significant Difference (HSD) test was used as a post hoc analysis following ANOVA. This method controls for the family-wise error rate and is suitable for pairwise comparisons between group means. The test was applied to data obtained at 50 days of degradation in both compost and seawater media, with a significance threshold of *p* < 0.05. Differences identified by the Tukey test were considered significant and are discussed in the results section.

## 3. Results

### 3.1. Weight Loss Assessment in Compost and Seawater

The thermal, morphological, and structural characterizations of the polymer blends revealed enhanced thermal stability as a result of increased addition of CA. This addition also led to reduced particle size and improved component interactions, by minimizing phase segregation and promoting blend homogeneity. As a result, a stable blend with a smoother surface and improved morphology was obtained [27]. At the end of the incubation period, progressive weight loss was observed, which continued over time, resulting in fragmentation and decreased thickness.

**Figure 2:**
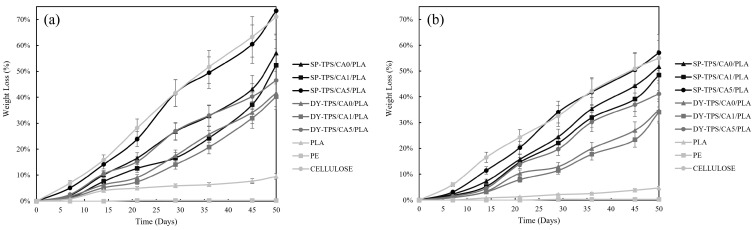
Weight loss of the TPS/CA/PLA blends in (**a**) compost and (**b**) seawater environments.

The weight variation as a function of time for the blends incubated in compost is illustrated in Figure 2a. Cellulose exhibited a weight loss of 71.04%, which is consistent with the results reported by Mantía et al. [34]. Values close to 67% were considered positive references. Conversely, the weight loss of the negative reference (PE) did not exceed 0.3% after 50 d. Since PE takes hundreds of years to degrade, this minimal loss confirms that the composting environment remained unaltered and did not interfere with the degradation process, thereby providing appropriate conditions for the development of the experiment.

All SP blends were evaluated in a compost environment. The SP-TPS/CA5/PLA blend reached the highest weight loss, reaching 73.41% at the end of 50 d, slightly surpassing that of cellulose in the final stage. In addition, it exhibited greater fragmentation and brittleness than the other blends. The SP-TPS/CA0/PLA blend achieved a weight loss of 57.05%, while SP-TPS/CA1/PLA was the least degradative of this group, with a weight loss of 52.34%. A similar trend was observed in the DY blends. DY-TPS/CA5/PLA exhibited the highest degradability, with a weight loss of 46.52%, followed by DY-TPS/CA0/PLA. Finally, DY-TPS/CA1/PLA was the least degraded among all blends buried in compost, with a weight loss of 40.20%. It also remained the most stable and least brittle throughout its degradation process.

Conversely, pure PLA degrades faster than conventional plastics such as PE; however, the incorporation of TPS and CA into PLA significantly accelerated its degradation in compost, with an approximate increase in weight loss of 40–60% for SP blends and 30–37% for DY blends, respectively. Previous studies [23,35] have shown that a CA graft enhances degradation by promoting the breakage of glycosidic bonds in the starch structure. This caused a high concentration of CA to increase the hydrolysis of the TPS chains, leading to faster degradation through acidolysis. This process is facilitated by improved component integration, as compatibilization enhances the interfacial interaction between phases [36].

The results obtained for weight loss at the seawater interphase are shown in Figure 2b. Cellulose, used as a positive reference, lost 55.05% of its initial weight, whereas PE, used as a negative reference, showed no significant degradation compared to the incubated blends, reaching a final weight loss of 0.2%. Similar to the results observed in compost, SP-TPS/CA5/PLA exhibited the highest weight loss in seawater, reaching 57.13% after 50 d and showing visible fragmentation from the second week of degradation. SP-TPS/CA0/PLA showed the second highest weight loss (51.67%), followed by SP-TPS/CA1/PLA, which had the lowest degradation among the SP blends, with a weight loss of 48.49%, and exhibited reduced brittleness and minimal color change. The DY blends followed a similar trend in relation to CA composition, as the blend with 5% (DY-TPS/CA5/PLA) exhibited the highest weight loss within this group. This was followed by DY-TPS/CA0/PLA, denoting DY-TPS/CA1/PLA as the least degradative of all blends, with a final weight loss of 34.06% [37].

These results are in line with previous studies showing that PLA degrades slowly under natural conditions, often requiring over 300 days to fully degrade at 25 °C [38]. By incorporating TPS and CA, substantial degradation was achieved within 50 days, particularly in the SP-TPS/CA5/PLA blend. This supports earlier findings by Ibrahim et al., who showed that CA enhances matrix disintegration by improving interfacial bonding and facilitating hydrolytic breakdown of the starch phase [35]. Notably, unlike prior work conducted under industrial composting, this study demonstrates that similar degradation effects can be achieved under ambient compost and marine conditions.

A possible explanation for this behavior can be attributed to the -OH groups in the blends. It has been demonstrated that after degradation, a decrease in the spectral signals associated with this functional group is observed [39]. This has an impact on degradation, since -OH groups are able to hydrolyze polymers in a faster way [40]. In the studied blends, DY-TPS/CA1/PLA and SP-TPS/CA1/PLA exhibited the lowest hydroxyl group signal, and SP-TPS/CA5/PLA and DY-TPS/CA5/PLA showed the highest intensity. This is linked to the molecular distribution following crosslinking with CA, suggesting that high amounts can enhance degradation, but minimal ratios might have a lagging effect [27].

Moreover, the enhanced degradation observed in blends with 5% CA can also be attributed to the synergistic interactions between CA, TPS, and PLA. CA acts as a crosslinker, chemically modifying -OH groups in starch and improving interfacial adhesion between the hydrophilic TPS and hydrophobic PLA [27]. This leads to a more homogeneous microstructure, reducing large phase domains and enhancing water diffusion and microbial accessibility [41]. Additionally, acidolysis promoted by CA contributes to the breaking of glycosidic bonds in starch, accelerating hydrolysis [42]. Meanwhile, TPS degrades first, leaving behind PLA-rich residues.

### 3.2. Biodegradability Assessment Based on CO_2_ Evolution

**Figure 3:**
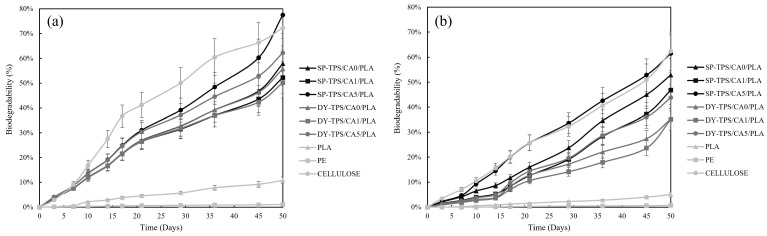
CO_2_ evolution curves showing biodegradability of TPS/CA/PLA blends in (**a**) compost and (**b**) seawater.

The biodegradability of the blends was analyzed by CO_2_ evolution for 50 d in both compost and seawater environments. CO_2_ was also detected in the blanks, confirming the presence of active microorganisms within the inoculum. Figure 3a,b illustrate the variation in biodegradability over time in compost and seawater, respectively.

In seawater, cellulose exhibited a maximum biodegradability of 62% at the end of 50 d. Meanwhile, in compost, the positive reference reached a maximum biodegradability of 72%, with a short lag phase during the first 7 d. This may be attributed to the incomplete adaptation of microorganisms to the environment during this time, resulting in lower CO_2_ emissions compared to later days of compost incubation [30]. Previous studies have reported biodegradability values of 70%–80% for cellulose and, in some cases, even higher under elevated temperature conditions [24,30,43]. These findings further support the effectiveness of the flasks in facilitating aerobic microbial activity, aided by the aeration provided by the perlite substrate in the compost. In contrast, PE, used as a negative reference, produced minimal CO_2_ emissions in both environments, as previously reported by Li et al. [44].

**Figure 4:**
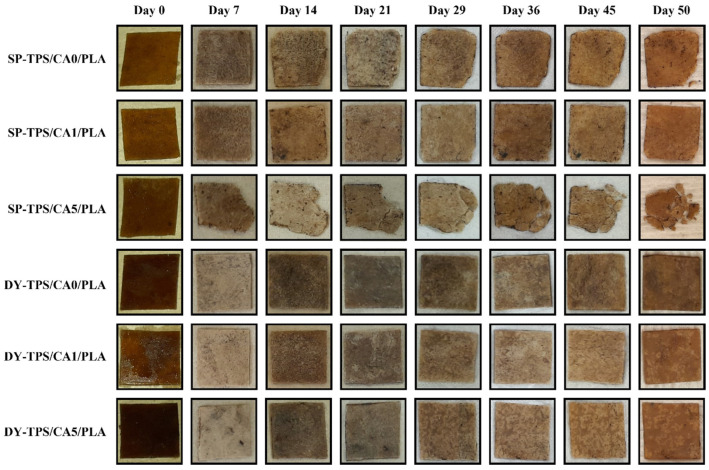
Visual degradation of TPS/CA/PLA blends in compost until day 50.

The buried blends in compost exhibited behavior consistent with the weight loss results. The blend of 5% CA (SP-TPS/CA5/PLA) showed the highest biodegradability, reaching 77% by the end of the 50-day period, with visible fragmentation after day 7, as shown in Figure 4. For the DY-TPS blends, a similar trend was observed, though with lower biodegradability values. Among these, the DY-TPS/CA5/PLA blend was the most degradable, followed by DY-TPS/CA0/PLA. Finally, DY-TPS/CA1/PLA was the most stable of all compost-incubated blends, with a biodegradability of 52%. Similar results have been reported for the anaerobic degradation of TPS/PLA blends, where a formulation with a 70% TPS ratio reached 60% biodegradability in a 30 d period under industrial composting conditions. These findings underscore the role of temperature in accelerating degradative processes and highlight the lagging effect of PLA, which can slow starch degradation due to its encapsulating effect on TPS molecules [45].

However, since the experiments were terminated at 50 d, it is not possible to determine whether degradation would have reached a plateau phase or continued at a reduced rate right after this point.

Generally, an insignificant lag phase was observed [46], followed by a prominent biodegradation phase, attributed to the adaptation of microorganisms to the compost inoculum. In SP blends, the degradation rate was mostly influenced by starch, which had a particle size of 9.5 μm, which is smaller than the 25.5 μm observed in DY blends. This suggests that, for a stable microbial population, larger starch granules require a longer incubation time to be completely metabolized (causing a delay). In contrast, smaller particles can be easily synthesized, leading to increased CO_2_ emissions and greater weight loss over a shorter period, as they are more susceptible to microbial degradation [26,30].

**Figure 5:**
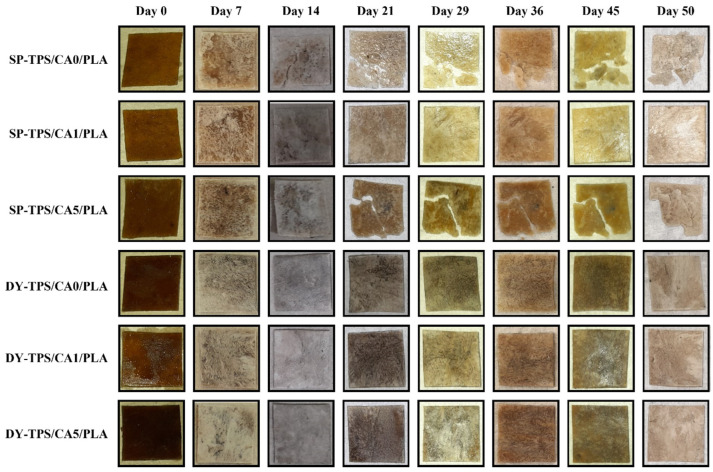
Visual degradation of TPS/CA/PLA blends in seawater until day 50.

**Figure 6:**
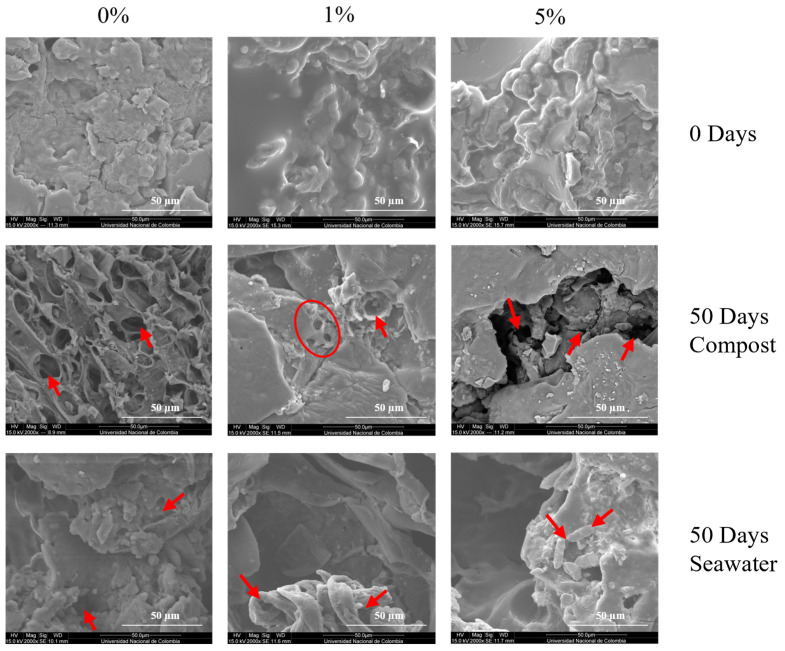
SEM micrographs of the SP-TPS/CA/PLA blends at 2000× before and after degradation in compost and seawater.

Figure 3b shows the variation in seawater biodegradability over time. Similar to the results in the compost environment, among the SP-TPS/CA/PLA blends, SP-TPS/CA5/PLA exhibited the highest biodegradability, displaying behavior comparable to that of cellulose, with visible fractures and disintegration from the second week, as shown in Figure 5. In contrast SP-TPS/CA0/PLA was the second most degradable blend, which exhibited fractures from the second week and reached a biodegradability of 52%, with ruptures from day 14. Consequently, SP-TPS/CA1/PLA was the most stable within this group, with a final biodegradability of 46%. Finally, SEM images (see Figure 6) reveal that SP-TPS/CA5/PLA exhibited more pronounced surface roughness, deeper pores, and micro-cracking than the other blends. The higher CA content promotes partial acidolysis and crosslinking within the starch phase. This acidolysis also reduces starch granule size and increases exposure of susceptible sites to enzymatic attack, accelerating hydrolysis [47]. Meanwhile, SP starch has a higher proportion of amorphous regions (amylose content), which facilitates water infiltration and microbial colonization, leading to faster disintegration and greater weight loss as the CA amount increases in SP starch blends.

The DY-TPS/CA/PLA blend also exhibited degradation behavior similar to the SP-based blends, but with lower biodegradability values. The DY-TPS/CA5/PLA blend was the most degradable, although its degradation trajectory differed significantly from that of cellulose. This was followed by DY-TPS/CA0/PLA, while DY-TPS/CA1/PLA was the least degradable of all the blends tested in seawater, exhibiting only small surface cracks compared to the rest of the analyzed blends. These results demonstrated faster degradation in compost than in seawater environments, which is supported by the fact that aerobic microorganisms are less commonly found in marine environments than in compost [48]. In addition, slow degradation by PLA hydrolysis starts at temperatures of 30 °C. In the infralittoral zone, this environmental condition is typically not observed. However, this degradation process can be improved by adding agitation to the flasks to improve oxygenation and wave simulation, keeping the phase difference [37].

TPS is highly hydrophobic, and the amorphous domains are quickly attacked by microorganisms in both compost and marine environments. This leads to micro-voids, which increase the surface area available for microbial colonization and facilitate overall degradation, evidencing cracks and fragmentation [49].

### 3.3. Morphological Characterization

#### 3.3.1. Morphological Changes After Degradation in Compost

The morphologies of the blends were studied by SEM. Figure 6 shows micrographs of the SP blends before and after 50 d of incubation in both the compost and seawater environments. At the end of the test, SP-TPS/CA0/PLA exhibited high surface roughness compared to the initial state, where the absence of CA promoted the growth of microorganisms because of TPS hygroscopicity and low affinity between the phases, which prevented the uniform distribution of the components, thereby placing this blend as the second most degraded within the group of those buried in compost. SP-TPS/CA1/PLA showed the presence of several holes but exhibited less roughness on the surface, indicating the occurrence of degradation in a lower proportion than that in the rest of the SP blends buried in compost. Conversely, SP-TPS/CA5/PLA exhibited major fragmentation, with greater surface deterioration in both length and depth. This indicates more extensive degradation, considering that a large part of the rough matrix observed at the beginning of the degradation has disappeared, indicating that this blend was the most degraded in the experiment, since it was the most susceptible to decomposition due to its higher -OH peak on the FTIR spectrum [27,40].

**Figure 7:**
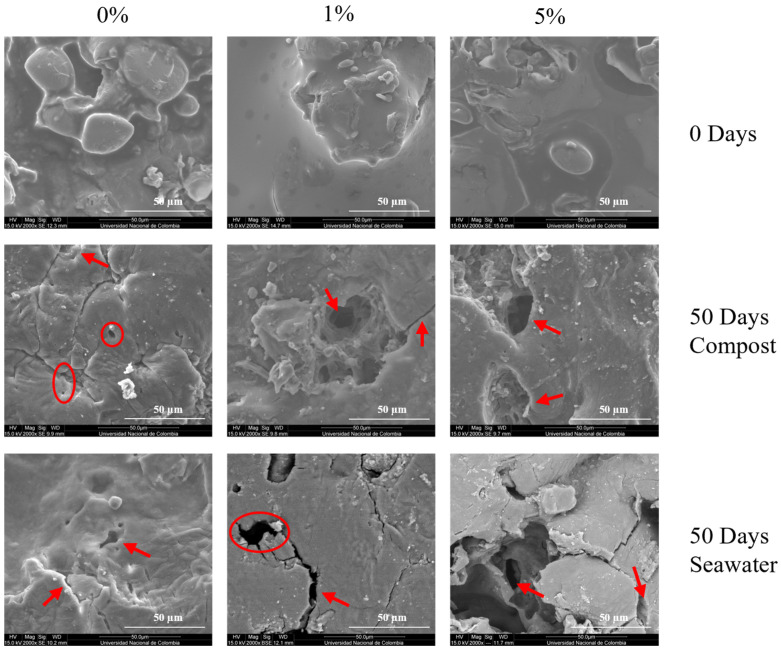
SEM micrographs of the DY-TPS/CA/PLA blends at 2000× before and after degradation in compost and seawater.

Similarly, Figure 7 shows the DY-TPS/CA/PLA blend surface, where less degradation was observed at the end of the incubation than that in the SP-TPS/CA/PLA blends. DY-TPS/CA0/PLA exhibited a lower proportion of roughness and cracks than the SP blends; however, it still indicated degradation. This is because most of the agglomerations present on day 0 were no longer visible, as they had been degraded by microorganisms. The DY-TPS/CA1/PLA blend exhibited degradation due to the presence of holes and small cracks, and the surface roughness was lower than that of the other blends in this group. Finally, the structure of DY-TPS/CA5/PLA was greater than those of the two previously mentioned blends because of the increase in roughness and the presence of a greater number of marked perforations. Furthermore, the agglomerations present on day 0 have disappeared, with indications of only the presence of some granules smaller than in DY-TPS/CA1/PLA. This means that their metabolism was faster because of the addition of CA as a crosslinker, which promoted degradation in high amounts due to acidolysis [35].

These findings align with Zhang et al. [50], who observed that higher amylose content in starch leads to increased surface roughness and more advanced biodegradation in blends. The more porous and fragmented structures seen in SP-based blends, compared to DY-based blends, reflect this difference in starch composition. Additionally, prior studies have shown that amylopectin-rich starches slow degradation due to higher crystallinity [51], similar to the more compact structure observed in the DY-TPS blends.

#### 3.3.2. Morphological Changes After Degradation in Seawater

The blends immersed in seawater were also analyzed using SEM. Micrographs of the SP-TPS/CA/PLA blends after 50 d of seawater incubation are shown in Figure 6. Initially, at 2000×, SP-TPS/CA0/PLA exhibited surface damage and roughness, indicating ongoing degradation. However, no visible cracks were observed, as in the compost incubation. Instead, the same agglomerations present on day 0 remained visible, suggesting limited degradation in this medium. In the SP-TPS/CA1/PLA blend, a deep hole, minimal roughness, and minor structural damage compared to the other blends were noticeable. These features confirmed the lower biodegradability of this blend among SP-TPS in seawater. SP-TPS/CA5/PLA displayed visible cracks, deep holes, and surface roughness produced by microbial activity. The presence of citric acid facilitated microbial attack during metabolism [46], making this blend the most susceptible to degradation in seawater.

Micrographs of the DY-TPS/CA0/PLA blend are shown in Figure 7, where the surface roughness, holes, and depressions indicate microbial activity. Additionally, the absence of agglomerations observed on day 0, along with the presence of visible starch granules, suggests delayed degradation of this type of raw material. DY-TPS/CA1/PLA exhibited holes and cracks, with reduced roughness, indicating that this blend underwent the least degradation. Finally, DY-TPS/CA5/PLA exhibited prominent holes, cracks, and roughness, along with a fibrous morphology, indicating the degradation of the blend into smaller particles [30]. This was further evidenced by the reduction of starch granules that generated roughness at the beginning of the incubation.

Consequently, the blends subjected to DY-TPS after 50 d showed less degradation than those subjected to SP-TPS. And the most degradative were SP-TPS/CA5/PLA and DY-TPS/CA5/PLA, respectively; since both blends have the same component ratio, the key distinction is the starch’s molecular composition and granular architecture. SP has a comparatively lower amylopectin content (54%) and smaller granule size, which increases the blend’s overall amorphous fraction [26]. Marine microorganisms can more readily enzymatically hydrolyze these amorphous domains [52], leading to micro-cracks and faster mass loss. DY starch, richer in amylopectin (74.4%) [26], retains more crystalline regions that are less susceptible to immediate hydrolysis, thus slowing overall degradation [30]. Additionally, the slightly larger granule size of DY starch may reduce the available surface area for colonization, further contributing to its relatively slower degradation in seawater.

### 3.4. Thermal Stability and Degradation Behavior of TPS/CA/PLA Blends

**Figure 8:**
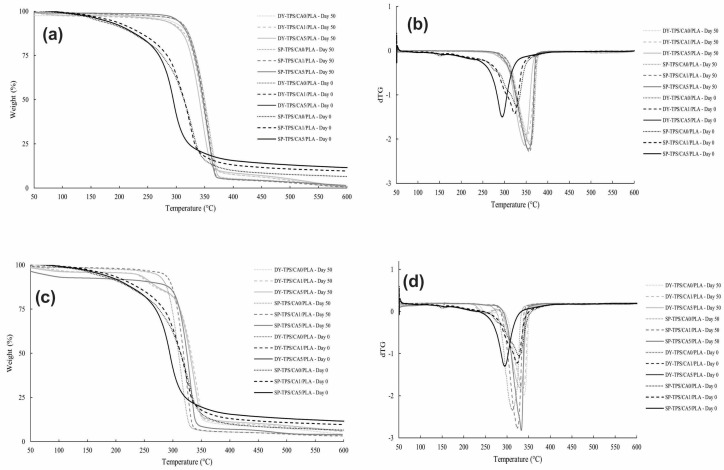
TGA and dTG of TPS/CA/PLA blends before and after 50 days of degradation in (**a**,**b**) compost and (**c**,**d**) seawater.

The thermal characterization of the blends was performed using TGA. Figure 8 illustrates the first derivatives of the thermograms and corresponding weight loss profiles for the TPS/CA/PLA blends as functions of temperature, both before and after compost incubation (Figure 8a,c). Before degradation, the blends exhibited mass loss in three steps. The first of these steps occurred from 51 to 180 °C due to water loss. The second step occurred in the range of 180–260 °C due to the added glycerol decomposition and other volatile compounds [53], while the third took place from 260 to 375 °C due to the weight loss of starch and PLA [26,54]. These degradation steps are represented by peaks in the derivative curves, with maximum degradation temperatures of 326.60, 323.89, and 294.75 °C at 0%, 1%, and 5% CA ratios, respectively.

For the blends containing SP-TPS, the curves show different weight losses after 50 d of incubation. The SP-TPS/CA0/PLA blend showed initial thermal degradation in the range of 35–62 °C, a second step from 328 to 366 °C, and a residue percentage of 1.02%. Similarly, the SP-TPS/CA1/PLA blend exhibited a first step from 35 to 53 °C, followed by degradation from 328 °C to 366 °C, with a residue percentage of 0.77%. The SP-TPS/CA5/PLA blend presented thermal decomposition from 34 to 57 °C and from 325 to 363 °C, with a residue percentage of 1.6%. The DY-TPS/CA0/PLA blend exhibited degradation from 327 to 370 °C, with 0.84% residues, while DY-TPS/CA1/PLA exhibited thermal degradation from 43 to 88 °C. For this blend, the second step occurred from 321 to 366 °C, generating residues of 0.59%, and the last step occurred from 498 to 536 °C. Similarly, evidence of the first stage of the DY-TPS/CA5/PLA blend was obtained in the interval of 43–87 °C, thus exhibiting the highest degradation reflected in the second step from 319 to 360 °C. Finally, the last step was observed from 484 to 553 °C, leaving 0.8% of the initial weight as residue at 600 °C. The initial loss temperatures (25 to 70 °C) correspond to moisture evaporation and the breakage of weak hydrogen bonds. Conversely, the second step (250 °C–375 °C) reflects the degradation of PLA and starch after 50 d of incubation. The final degradation stage (400–500 °C) is associated with the calcination and oxidation of residual polymeric components [53,55].

Analysis of the derivative thermogravimetric curves (dTG) after 50 d showed thermal degradation peaks near 350 °C, consistent with the literature values for the characteristic range of PLA degradation in its pure state [54]. These results suggest the previous degradation of certain components of the blends during incubation (e.g., a certain part of water, glycerol, and mainly starch) due to their higher degradation capacity. As a result, the post-incubation blends were predominately composed of PLA, which degrades more slowly than the other components. This further explains the similarity in the shapes of the peaks obtained in the thermogram derivatives and their greater thermal stability compared to their behavior at day 0, as also reported in previous studies [53,54]. Although PLA is a biodegradable polymer, its limited degradation in this study is likely due to the room-temperature conditions used, as PLA typically requires temperatures of at least 60 °C to undergo effective hydrolysis. Hence, higher degradation percentages could likely be achieved under industrial composting conditions [56].

The thermal degradation of the blends incubated in seawater was also evaluated by TGA. Figure 8c,d display the weight losses vs. temperature for the TPS/CA/PLA blends. For the SP-TPS blends, SP-TPS/CA0/PLA exhibited two main degradation stages: the first from 35 to 57 °C, followed by another step from 290 to 313 °C, with a residue of 3.56%. SP-TPS/CA1/PLA showed initial degradation from 35 to 76 °C, followed by a second step from 300 to 323 °C, with residues of 3.22%. Finally, SP-TPS/CA5/PLA exhibited weight loss from 35 to 27 °C and then from 311 to 331 °C, resulting in 4.08% residues. DY-TPS blends incubated in seawater exhibited different thermal degradation behavior compared to the SP-based blends. DY-TPS/CA0/PLA exhibited weight loss from 52 to 108 °C, followed by a second step from 240 to 260 °C and a third step from 317 °C to 348 °C, with residues of 4.3%. DY-TPS/CA1/PLA had an initial thermal degradation from 63 to 114 °C, peak degradation from 235 to 257 °C, and then a peak from 317 to 325 °C, with 5.8% residues. Finally, DY-TPS/CA5/PLA exhibited initial weight loss from 48 °C to 91 °C, followed by a minor peak from 239 to 261 °C and a final step from 314 to 347 °C, with residues of 6.13%.

The compost degradation behavior of the SP-TPS/CA/PLA blends in seawater was observed to be similar. The initial weight loss observed in the first degradation steps corresponded to water losses due to weak and strong bond breakage ranging from 25 to 70 °C and from 70 to 180 °C. The degradation second step occurred within the degradation range of the PLA–starch blend close to 320 °C [53], indicating that PLA remained the dominant component in most blends, as it requires higher temperatures to degrade [56]. This implied that the compatibilizers and part of the starch were degraded by aerobic microorganisms, depending on the composition and compatibility of the blend [54]. Conversely, the DY-based blends showed two degradation peaks in the dTG. The higher-temperature peaks (346 to 342 °C) confirmed the continued presence of PLA and TPS in the matrix. The lower peaks (260 °C) might be attributed to the presence of glycerol [53], indicating a lower degradation in DY-TPS/CA1/PLA than the rest of the blends because of the greater amount of glycerol in its molecular structure after 50 d of degradation with incomplete phase compatibility, which supports the analysis results of biodegradability by CO_2_ evolution, which has also indicated reduced biodegradability for this blend.

The variation in the residue content after incineration was associated with biodegradability and the degradative medium. Blends that underwent greater degradation exhibited a higher residue percentage compared to those less affected by the environment. This was attributed to the increased absorption of inorganic compounds present in the degradative medium. As degradation progressed, the blends gained porosity, allowing greater uptake of these compounds, which remained as ash after the incineration [30]. TOC analyses of the media showed that there was a larger amount of organic matter in compost than in seawater, which promoted a lower availability of inorganic compounds in the compost. This further explains why the blends in seawater exhibited a higher number of residues than those buried in compost.

### 3.5. Statistical Analysis Results

#### 3.5.1. Analysis of Variance at 21 Days

**Figure 9:**
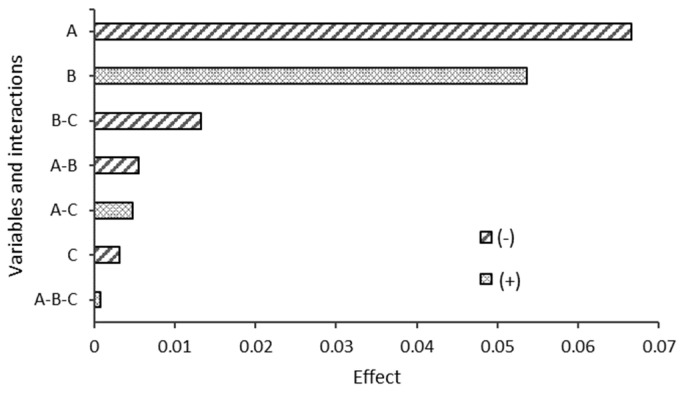
ANOVA analysis after 21 days.

Figure 9 shows the effects of starch type (variable A), CA ratio (variable B), and degradation environment (variable C) on the biodegradability of the blends obtained by ANOVA after 21 d of degradation. The starch type was the most influential variable in the biodegradability of the blends at 21 d. The results show that starch type had a negative effect on biodegradability, indicating that blends with SP starch exhibited higher biodegradability than those with DY starch. The *p*-value for this variable is 0.0006 (*p* < 0.05), confirming that its influence is statistically significant. This suggests that SP starch is more susceptible to degradation than DY starch in the short term.

The CA ratio was also found to have a statistically significant effect on biodegradability after 21 d, with a *p*-value of 0.0162 (*p* < 0.05). The calculated positive effect indicated that higher CA ratios resulted in higher blend biodegradability. This finding highlights the importance of CA as a crosslinking agent that can accelerate the degradation of blends.

The degradation environment had a negative effect on biodegradability after 21 d, implying that blends in compost had a lower average biodegradability than blends in seawater. However, its *p*-value of 0.8569 (*p* > 0.05) indicated that the difference was not statistically significant, suggesting that the degradation medium did not have a considerable influence on biodegradability during this period. Finally, the interactions between starch type, CA ratio, and degradation medium did not show a statistically significant influence on biodegradability at 21 d, with all *p*-values > 0.05. This suggests that, in the short term, the variables act independently without significantly affecting the biodegradability of the blends in combination. This effect may be attributed to the fact that citric acid primarily influences the surface morphology of the material or acts during the initial stages of hydrolytic degradation. Once these more accessible regions are decomposed, the overall degradation rate decreases and becomes increasingly governed by other factors, such as polymer crystallinity, intermolecular interactions, and the intrinsic structural resistance of the bulk matrix.

#### 3.5.2. Analysis of Variance at 50 Days

**Figure 10:**
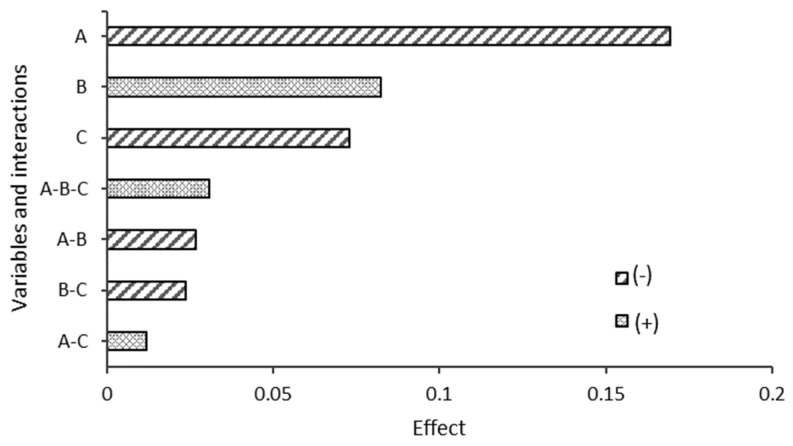
ANOVA analysis after 50 days.

The ANOVA results for the effects of the experimental variables on the biodegradability of the blends after 50 d are presented in Figure 10. After 50 d of degradation, the starch type continued to be the most influential variable, having a negative effect on biodegradability. Blends with SP starch showed higher biodegradability than the DY starch blends. The *p*-value for this variable was 0.0016 (*p* < 0.05), reaffirming its significant influence on biodegradability over time. In contrast to the results at 21 d, the CA ratio did not have a statistically significant effect on biodegradability at 50 d, with a *p*-value of 0.1677 (*p* > 0.05). Although the calculated effect remained positive, indicating that higher CA concentrations can increase biodegradability, its influence was not statistically significant over the long term. This could indicate the saturation of the effect of CA or the adaptation of the material to a degrading environment.

Similarly, the degradation medium had a negative effect on biodegradability at 50 d, similar to the results at 21 d. The compost blends showed lower average biodegradability. However, at a *p*-value of 0.1353 (*p* > 0.05), the difference between the degradation media was not statistically significant, suggesting that the influence of the medium stabilized over time. The interactions between the variables did not show a statistically significant influence on biodegradability at 50 d, with all *p*-values > 0.05. This indicates that, in the short term, the main variables act independently without significantly affecting biodegradability in combination.

#### 3.5.3. Comparison of Results Between 21 and 50 Days

Comparing the results of the ANOVA analysis after 21 and 50 d, it was observed that starch type remained the most influential variable on biodegradability in both periods. However, the CA ratio, which was significant at 21 d, did not retain its significance at 50 d, suggesting that its effect may be more noticeable in the early stages of degradation. In contrast, the degradation medium did not show a statistically significant difference in either period. The interactions between the variables did not have a significant influence on either analysis, implying that the studied variables had independent effects on the biodegradability of the blends.

#### 3.5.4. Tukey’s Multiple Comparisons Test in Seawater

**Figure 11:**
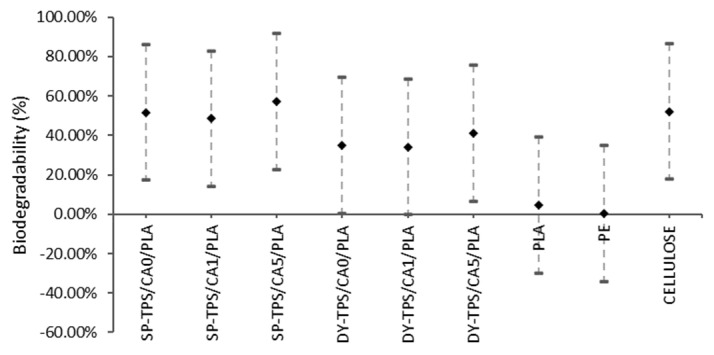
Tukey analysis in seawater.

The results of the Tukey analysis are presented in Figure 11, which demonstrates that all blends have similar biodegradability in seawater. In addition, the biodegradability of the blends was comparable to that of cellulose. However, significant differences were observed when the biodegradability of the blend was compared with that of PLA and PE. These results indicate that, in a marine environment, the studied blends exhibit degradation behavior comparable to that of cellulose and superior to that of conventional plastics such as PE.

#### 3.5.5. Tukey’s Multiple Comparisons Test in Compost

**Figure 12:**
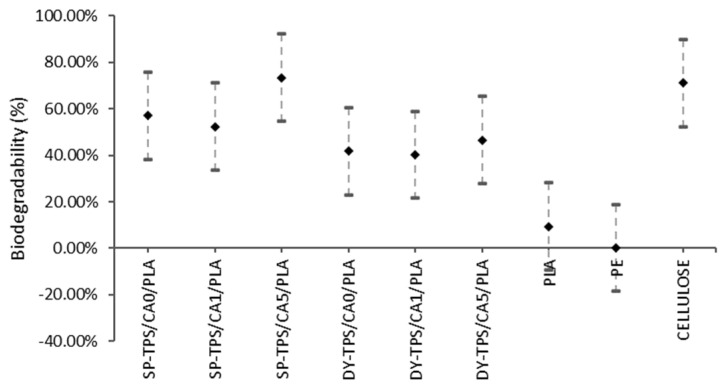
Tukey analysis in water and compost.

The Tukey analysis results of compost are presented in Figure 12, and specific differences were identified between the different blends.

- The SP-TPS/CA0/PLA and SP-TPS/CA1/PLA blends showed similar biodegradability, suggesting that the CA ratio did not have a significant impact on the degradability of blends with 0% and 1% CA in the compost.
- SP-TPS/CA5/PLA: This blend had the highest biodegradability, which is similar to that of cellulose. This suggests that a higher CA ratio (5%) significantly increased the degradability of the blends in the compost.
- The DY-TPS/CA0/PLA, DY-TPS/CA1/PLA, and DY-TPS/CA5/PLA blends with DY starch showed similar biodegradability regardless of the CA ratio. This indicates that in the case of DY starch, the variation in the CA ratio did not have a significant impact on the degradability of the compost.

In addition, the difference in the biodegradability of all the blends compared to that of PLA and PE persisted. Regardless of their specific formulations, the developed blends demonstrated significantly higher biodegradability than PLA and PE in the compost.

## 4. Conclusions

Overall, the TPS/CA/PLA blends with SP-TPS and DY-TPS exhibited faster degradation than conventional plastics (PE) and PLA. In seawater, the blends showed a similar behavior to cellulose biodegradation, whereas in compost, the CA ratio had a higher impact on blends with SP-TPS than on blends with DY-TPS. The most degradative blends contained 5% CA, followed by blends without CA. Blends with 1% CA were ranked as the most stable. However, all the blends required more than 50 d to fully degrade. Additionally, statistical analysis showed that the DY-TPS blends experienced slower degradation than the SP-TPS blends, indicating that the starch type and CA ratio are critical factors influencing the biodegradability of the blends, especially during the first weeks of incubation. SEM analysis revealed microbial activity in the blends, including structural damage after 50 d, supporting the occurrence of degradation. TGA results showed weight losses in the stages from 25 to 180 °C and from 260 to 365 °C corresponding to water evaporation and starch and PLA degradation, thus demonstrating a loss of glycerol in all blends submerged in seawater, except for DY-TPS blends. Therefore, the plausibility of TPS acting as a continuous matrix phase depends directly on the final applications of the composite. Finally, the degradation behavior of the blends mainly depends on the number of -OH groups, as they are mostly attracted by the oxygen present in the environment, thereby undergoing aerobic degradation. The observed synergy among starch type, CA content, and degradation conditions reinforces the potential of using underutilized native starches as sustainable feedstocks for biodegradable composites under natural and marine environments.

## Figures and Tables

**Table 1:** Composition of the TPS/CA/PLA blends.

Blend	TPS (%)	PLA (%)	CA (%)
DY-TPS/CA0/PLA	60.0	40.0	0.0
DY-TPS/CA1/PLA	59.4	39.6	1.0
DY-TPS/CA5/PLA	57.0	38.0	5.0
SP-TPS/CA0/PLA	60.0	40.0	0.0
SP-TPS/CA1/PLA	59.4	39.6	1.0
SP-TPS/CA5/PLA	57.0	38.0	5.0

**Table 2:** Compost characterization.

Parameter	Value
Humidity (%) *	6.00
Ash (%) *	48.90
Total Organic Carbon [TOC] (%) *	18.60
Total Nitrogen	1.56
Assimilable Phosphorus [P_2_O_5_] (%) *	3.20
Water-soluble Potassium [K_2_O] (%) *	1.00
pH	6.50
Density (g/cm^3^)	0.46
Cation Exchange Capacity (meq/100 g) *	71.30
Humidity Retention Capacity (%) *	149.0
Dry Solids (%)	55.00
Volatile Solids (%)	53.00
C/N Ratio	11.92

* Parameter extracted from the compost SDS.

**Table 3:** Characterization of seawater.

Parameter	Value
Salinity (ppt)	30.75
Acidity	-
Alkalinity [CaCO_3_] (mg/L)	105.0
Carbon Dioxide [CO_2_] (mg/L)	25.00
Nitrite [NO_2_-N] (mg/L)	<0.66
Nitrate [NO_3_-N] (mg/L)	<10.0
Phosphate [PO_4_] (mg/L)	5.00
Dissolved Oxygen [O_2_] (mg/L)	6.00
Density (g/cm^3^)	1.014
pH	7.06
Total Organic Carbon [TOC] (mg/L)	112.95
Conductivity (mS/cm)	45.80
Temperature (°C)	27.00

**Table 4:** Setup of the statistical design of the experiments.

Variable	Levels	Coded Variables
Type of starch (A)	SP	−1
	DY	1
CA concentration (B)	0%	−1
	1%	0
	5%	1
Degradation medium (C)	Compost	−1
	Seawater	1

## Data Availability

The original contributions presented in this study are included in the article. Further inquiries can be directed to the corresponding author.
